# Zearalenone Induces Gap Junction Damage in Ovine Ovarian Granulosa Cells by Upregulating GPR30 and Activating the Oxidative Stress–NLRP3 Inflammasome Axis

**DOI:** 10.3390/biom16060837

**Published:** 2026-06-07

**Authors:** Xiaoyun Pang, Dong Zhang, Hongwei Duan, Zhenxing Yan, Xianghong Du, Lujie Zhao, Jincheng Yang, Li Xue, Yanyan Wang, Yuxuan He

**Affiliations:** 1 College of Veterinary Medicine, Gansu Agricultural University, Lanzhou 730070, China; 1073323020128@st.gsau.edu.cn (X.P.); zhangd@st.gsau.edu.cn (D.Z.); 1073323010069@st.gsau.edu.cn (Z.Y.); duxiangh@gsau.edu.cn (X.D.); 1073323020129@st.gsau.edu.cn (L.Z.); 1073324120274@st.gsau.edu.cn (J.Y.); 1073324020130@st.gsau.edu.cn (L.X.); 1073324120280@st.gsau.edu.cn (Y.W.); 2 Gansu Key Laboratory of Animal Generational Physiology and Reproductive Regulation, Lanzhou 730070, China; 3 College of Veterinary Medicine, Anhui Agricultural University, Hefei 230036, China; duanhw@ahau.edu.cn

**Keywords:** zearalenone, ovarian granulosa cells, GPR30, NLRP3 inflammasome, gap junction

## Abstract

Ovarian granulosa cells (GCs) ensure proper follicular development and oocyte maturation through gap-junction-mediated intercellular communication. Zearalenone (ZEA), a mycotoxin with estrogen-like activity, specifically targets and impairs ovarian function. Most existing studies have focused on ZEA-induced apoptosis in GCs, but whether ZEA disrupts gap junctions in ovarian GCs remains unclear. Therefore, the aim of this study was to investigate whether and how ZEA induces gap junction injury in ovine ovarian GCs, with a particular focus on the roles of G protein-coupled receptor 30 (GPR30), oxidative stress, and the NLRP3 inflammasome. In the present study, primary ovine ovarian GCs were isolated, cultured, and treated with different concentrations of ZEA to establish a gap junction injury model, and specific inhibitors/antagonists were used to investigate the underlying mechanisms. The results showed that ZEA decreased granulosa cell viability and significantly inhibited the expression of the gap junction proteins Connexin 43 (Cx43) and Connexin 37 (Cx37) in a concentration-dependent manner. ZEA treatment also significantly upregulated the expression of the NOD-like receptor familypyrindomain containing 3 (NLRP3) inflammasome-related proteins (NLRP3, ASC, Cleaved Caspase-1, and the downstream pro-inflammatory cytokine IL-1β) in a concentration-dependent manner. Pretreatment with the NLRP3-specific inhibitor MCC950 significantly reversed ZEA-induced downregulation of Cx43 and Cx37 and effectively blocked NLRP3 inflammasome activation, indicating that NLRP3 is a key target in ZEA-induced gap junction injury. Further experiments confirmed that ZEA treatment significantly increased oxidative stress levels in granulosa cells; pretreatment with the reactive oxygen species (ROS) scavenger N-acetylcysteine (NAC) restored the ZEA-induced downregulation of Cx43 and Cx37 and suppressed NLRP3 inflammasome activation, suggesting that ROS acts as an upstream regulator of NLRP3 inflammasome activation. Moreover, ZEA treatment altered GPR30 expression levels, and pretreatment with the GPR30 antagonist G15 effectively inhibited ZEA-induced ROS production, NLRP3 inflammasome activation, and downregulation of Cx43/Cx37, indicating that ZEA exerts its effects through functional activation of GPR30. Collectively, ZEA activates the GPR30 receptor, induces ROS accumulation in granulosa cells, and subsequently triggers NLRP3 inflammasome activation, ultimately leading to downregulation of Cx43 and Cx37 and gap junction dysfunction. This study reveals a previously unrecognized molecular mechanism by which ZEA induces gap junction injury in ovarian GCs, providing potential therapeutic targets and a theoretical basis for preventing ZEA-induced ovarian dysfunction and improving animal reproductive health.

## 1. Introduction

The rapid development of modern industry and agriculture has enhanced production efficiency, but it has also led to increasingly severe environmental pollution, posing continuous threats to ecosystems, plants, animals, and human health [1]. In animal husbandry, mycotoxin contamination has become a key risk factor affecting animal health and production efficiency due to its concealment, pervasiveness, and high toxicity [2,3]. Zearalenone (ZEA), an estrogenic mycotoxin produced by *Fusarium species*, is widely present in grains such as corn and wheat [4]. Due to its chemical structure resembling endogenous estrogens, ZEA can competitively bind to estrogen receptors and exert estrogen-like activity, making it one of the most harmful mycotoxins to the reproductive systems of female animals [5,6,7,8]. ZEA exposure can cause a range of reproductive disorders in female animals, including abnormal estrus, reduced conception rates, miscarriages, and impaired offspring performance, leading to significant economic losses in animal husbandry [9].

As an economically important livestock species and a sentinel for environmental health, sheep are central to animal husbandry, with their reproductive performance directly influencing production efficiency [10,11,12]. In female mammals, the ovary serves as a key reproductive organ, wherein granulosa cells (GCs), the most abundant and functionally essential somatic cells within ovarian follicles, orchestrate follicular development, steroidogenesis, and the paracrine support of oocyte maturation [13,14]. Intercellular communication among GCs and between GCs and the oocyte is mediated by gap junctions composed of connexin proteins, which are crucial for the exchange of metabolites and signaling molecules, as well as for maintaining follicular microenvironmental homeostasis [15,16,17]. Accumulating evidence indicates that upon ingestion of ZEA contaminated feed, ZEA enters systemic circulation and selectively accumulates in the ovaries, where it preferentially targets GCs [18]. However, the precise molecular mechanisms by which ZEA disrupts the structural integrity of gap junctions in GCs, thereby leading to reproductive toxicity, have not yet been fully elucidated.

Oxidative stress is a core mechanism in environmental toxin-induced cellular damage [19]. Studies have shown that excessive accumulation of reactive oxygen species (ROS) can trigger GC apoptosis and is closely associated with the occurrence of follicular developmental disorders such as follicular cysts [20]. G protein-coupled receptor 30 (GPR30), a novel estrogen receptor localized in the cell membrane, rapidly responds to stimulation by estrogen-like compounds such as ZEA and initiates intracellular signaling transduction [21,22,23]. Furthermore, the NOD-like receptor familypyrin domain containing 3 (NLRP3) inflammasome, a critical hub linking oxidative stress and inflammatory responses, can be activated by signals such as ROS [24]. Although the NLRP3 inflammasome may disrupt gap junction communication by down-regulating connexin expression, its specific role in ZEA-induced ovarian damage remains to be elucidated [25].

Based on the above background, primary ovine ovarian GCs were cultured and treated with ZEA to establish a gap junction injury model. Cell viability, gap junction proteins (Connexin 43 (Cx43)/Connexin 37 (Cx37)), NLRP3 inflammasome activation, and ROS levels were assessed. Intervention experiments were conducted using MCC950 (NLRP3 inhibitor), NAC (ROS scavenger), and G15 (GPR30 antagonist). This study aimed to investigate whether ZEA damages gap junctions in ovine granulosa cells via the GPR30/ROS/NLRP3 signaling axis, thereby providing a theoretical basis and potential targets for preventing ZEA-induced ovarian injury.

## 2. Materials and Methods

### 2.1. Reagent and Antibodies

ZEA (purity ≥ 98.22%, Cat. No. HY-103447) was purchased from MedChemExpress (Waltham, MA, USA). G15 (a selective GPR30 antagonist, Cat. No. HY-103449, MedChemExpress), N-acetylcysteine (NAC, a reactive oxygen species scavenger, Cat. No. S0077, Beyotime Biotechnology, Shanghai, China), and MCC950 (a selective NLRP3 inflammasome inhibitor, Cat. No. HY-12815, MedChemExpress) were also purchased. The fluorescent probe 2′,7′-dichlorodihydrofluorescein diacetate (DCFH-DA, Cat. No. S0033) for ROS detection was supplied by Beyotime Biotechnology (Shanghai, China). Cell Counting Kit-8 (CCK-8, Cat. No. IV08-100T) for cell viability assessment was obtained from Invigentech (Irvine, CA, USA). Fetal bovine serum (FBS) and Dulbecco’s Modified Eagle Medium/Nutrient Mixture F-12 (DMEM/F12) medium were sourced from Gibco (Grand Island, NE, USA). Penicillin (100 IU/mL) and streptomycin (100 μg/mL) were obtained from Solarbio (Beijing, China). The enhanced chemiluminescence (ECL) substrate was acquired from Vazyme (Nanjing, China).

Primary antibodies against Cx43 (Cat. No. 26980-1-AP), NLRP3 (Cat. No. 19771-1-AP), ASC (Cat. No. 10500-1-AP), Cleaved Caspase-1 (Cat. No. 22915-1-AP), IL-1β (Cat. No. 26048-1-AP), and HRP-conjugated goat anti-mouse IgG (H+L) (Cat. No. SA00001-1) were obtained from Proteintech (Wuhan, China). Antibodies against Cx37 (Cat. No. YT1043) were purchased from Immunoway (Plano, TX, USA). GPR30 (Cat. No. bs-1380R) and β-actin (Cat. No. bs-0061R) primary antibodies, as well as Follicle-stimulating hormone receptor (FSHR, Cat. No. PA5-50963), were sourced from Bioss (Beijing, China). HRP-conjugated goat anti-rabbit IgG (H+L) (Cat. No. SA00001-2) was also obtained from Proteintech. For immunofluorescence staining, CoraLite488-labeled goat anti-rabbit IgG (Cat. No. SA00013-2) and CoraLite594-labeled goat anti-mouse IgG (Cat. No. SA00013-3) secondary antibodies were purchased from Proteintech (Wuhan, China). All other chemical reagents not explicitly mentioned were of analytical grade and obtained from standard commercial suppliers.

### 2.2. Experimental Materials

Ovaries were collected from adult, healthy female Small-tailed Han ewes (body weight, 25–30 kg) at a local slaughterhouse in Yongdeng County, Lanzhou City. A total of 30 ewes were used in this study. Ovaries were transported to the laboratory on ice within 4 h of collection. Only antral follicles ranging from 3–6 mm in diameter were selected for GC isolation. From these, a total of approximately 150 follicles were aspirated. To ensure biological variability, GCs from 5–6 follicles were combined to form a single sample, and all experiments were conducted with a minimum of three such independent sample sets.All animal experiments were conducted in accordance with the approval granted by the Animal Care and Use Committee of Gansu Agricultural University (No. GSAU-ETH-VMC 2023-021, approval date: 9 March 2023).

### 2.3. Cell Culture and Treatment

#### 2.3.1. Cell Culture

Fresh ovaries were collected from a local slaughterhouse, placed in sterile PBS pre-warmed to 37 °C in a thermostatic container, and transported to the laboratory within 4 h. All subsequent cell isolation procedures were performed on a clean bench. Follicular fluid containing GCs and oocytes was aspirated from antral follicles using a 1 mL syringe, washed twice with PBS, and then subjected to enzymatic digestion to dissociate GCs from oocytes. The isolated GCs were initially cultured in DMEM/F12 medium supplemented with 10% FBS for 24–48 h at 37 °C in a humidified atmosphere containing 5% CO_2_ to allow recovery. After this pre-culture period, cells were trypsinized, counted, and reseeded into appropriate culture plates. Following 12 h of attachment, cells were serum-starved in DMEM/F12 medium for 12 h prior to treatment. Prior to formal experimentation, the purity and identity of the cultured GCs were validated by immunofluorescence detection of the FSHR, a specific marker for GCs.

#### 2.3.2. Experimental Design

Prior to treatment, cells were serum-starved in DMEM/F12 medium for 12 h. To evaluate the cytotoxic effects of ZEA, GCs were treated with ZEA at final concentrations of 0, 20, 40, or 60 μM for 24 h, based on the CCK-8 results (see Section 2.4). For mechanistic studies, the following experimental groups were included: (1) control (vehicle only); (2) ZEA alone (60 μM, 24 h); (3) ZEA + MCC950 (10 μM MCC950 pretreatment for 2 h, then co-incubation with 60 μM ZEA for 24 h); (4) ZEA + NAC (5 mM NAC pretreatment for 2 h, then co-incubation with 60 μM ZEA for 24 h); and (5) ZEA + G15 (10 μM G15 pretreatment for 2 h, then co-incubation with 60 μM ZEA for 24 h). All pretreatments were performed without medium replacement prior to ZEA addition.

### 2.4. Cell Viability Assay

Cell viability of GCs was assessed using the CCK-8 assay according to the manufacturer’s protocol. Primary GCs were seeded at a density of 5 × 10^3^ cells/well in a 96-well plate. After 24 h of culture, cells were treated with different concentrations of ZEA (0, 10, 20, 40, 60, and 80 μM) for 24 h. The medium was then removed, and wells were added with medium containing 10% CCK-8 solution. The wells were incubated in a cell culture incubator for 3 h. The optical density (OD) value was measured at a wavelength of 450 nm. A wider concentration range (0–80 μM) was used to generate a complete dose–response curve and to determine the appropriate concentrations for subsequent mechanistic experiments.

### 2.5. Determination of Total ROS in GCs

Intracellular ROS levels were measured using DCFH-DA. Briefly, cells were seeded into 24-well plates at a density of 2 × 10^4^ cells/well and subjected to the indicated treatments. Following treatment, cells were incubated with 10 μM DCFH-DA in serum-free medium at 37 °C in the dark for 30 min, gently washed twice with PBS, and immediately imaged using a fluorescence microscope. Fluorescence intensity was quantified using ImageJ software (Version 1.54r, National Institutes of Health, Bethesda, MD, USA).

### 2.6. Western Blotting Assay

Cells were washed with ice-cold PBS and lysed in radioimmunoprecipitation assay (RIPA) buffer (Solarbio, Beijing, China) supplemented with 1% phenylmethanesulfonyl fluoride (PMSF, Solarbio, Beijing, China). Protein extraction was enhanced by three freeze–thaw cycles. Western blotting was then performed as previously described. Briefly, equal amounts of protein were separated by 10% sodium dodecyl sulfate-polyacrylamide gelelectrophoresis and transferred onto a 0.45 μm polyvinylidene difluoride membrane (Millipore, Billerica, MA, USA). The membrane was blocked with 5% bovine serum albumin (BSA) in Tris-buffered saline containing 0.1% Tween-20 for 2 h at room temperature. Subsequently, the membrane was incubated overnight at 4 °C with the following primary antibodies: anti-Cx43 (1:1000), anti-Cx37 (1:1000), anti-GPR30 (1:1000), anti-NLRP3 (1:1000), anti-ASC (1:1000), anti-Cleaved Caspase-1 (1:1000), anti-IL-1β (1:1000), and anti-β-actin (1:3000). After washing with TBST, the membrane was incubated with horseradish peroxidase (HRP)-conjugated goat anti-rabbit IgG (1:5000) for 1 h at 37 °C. Protein bands were visualized using an ECL substrate (Vazyme, Nanjing, China). Images were acquired using the Amersham Imager 600 chemiluminometer (GE Healthcare Bio-Sciences AB, Uppsala, Sweden). Band intensities were quantified using ImageJ software (National Institutes of Health, Bethesda, MD, USA), and target protein levels were normalized to β-actin as the loading control.

### 2.7. Statistical Analysis

All experiments were performed in at least three independent biological replicates. Data are presented as mean ± standard deviation (SD). Statistical analyses were carried out using GraphPad Prism 8.0.2 (GraphPad Software, San Diego, CA, USA), OriginPro 9 (OriginLab Corporation, Northampton, MA, USA), and ImageJ software (National Institutes of Health, Bethesda, MD, USA). Comparisons between two groups were performed using the independent samples Student’s *t*-test. Multiple group comparisons were conducted using one-way analysis of variance (ANOVA), followed by Tukey’s post hoc test for multiple comparisons. Statistical significance was defined as *p* < 0.05.

### 2.8. Use of Generative AI Tools

During the preparation of this manuscript, the authors used DeepSeek (DeepSeek-V3; available at https://deepseek.com) for the purposes of language polishing, grammar correction, sentence structure improvement, formatting, and punctuation. The authors have reviewed and edited all AI-generated output and take full responsibility for the content of this publication.

## 3. Results

### 3.1. Effects of ZEA on Cell Viability and Gap Junction Impairment in Ovine Ovarian GCs

**Figure 1:**
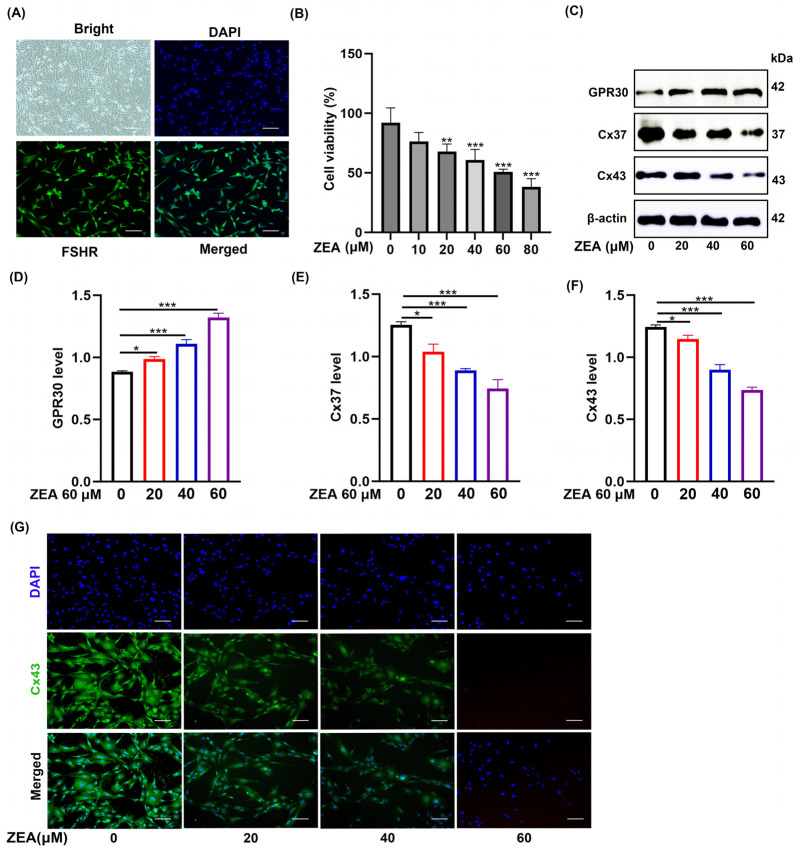
ZEA dose-dependently reduces Cx43 and Cx37 expression in GCs. (**A**) Immunofluorescence staining showing FSHR expression in GCs; Scale bar = 100 μm. (**B**) CCK-8 assay for cell viability in GCs treated with different concentrations of ZEA (*n* = 6). (**C**) Western blotting analysis of Cx37 and Cx43 protein levels after ZEA treatment (*n* = 3), with β-actin as internal control. (**D**,**E**) Quantitative analysis of Cx37 and Cx43 protein levels in GCs after ZEA treatment. (**F**,**G**) Immunofluorescence staining intensity and quantification of Cx43; Scale bar = 100 μm. The loading control was β-actin, and data are presented as mean ± SD. * *p* < 0.05, ** *p* < 0.01, *** *p* < 0.001. Original images can be found in Supplementary Materials.

First, the cultured cells were identified via immunofluorescence staining for the FSHR. Clear green fluorescence was detected on the cell membranes, verifying that the cultured cells were ovine ovarian GCs (Figure 1A). After treatment with ZEA (0–80 μM) for 24 h, the viability of the granulosa cells declined in a dose-dependent manner. Notably, the 10 μM ZEA treatment caused only a slight reduction in cell viability (<10%), while the 80 μM ZEA treatment led to excessive cytotoxicity (a reduction in viability of >70%, Figure 1B). Based on the combined considerations of cytotoxicity and dose effects, four concentrations (0, 20, 40, and 60 μM) were chosen for the subsequent mechanistic studies. Furthermore, the expression levels of the key gap junction proteins Cx43 and Cx37 were evaluated. The results demonstrated that ZEA treatment significantly and dose-dependently reduced the protein expression of Cx43 and Cx37 (Figure 1C–G).

### 3.2. ZEA Induces Activation of the NLRP3 Inflammasome Signaling Pathway in Ovine Ovarian GCs

**Figure 2:**
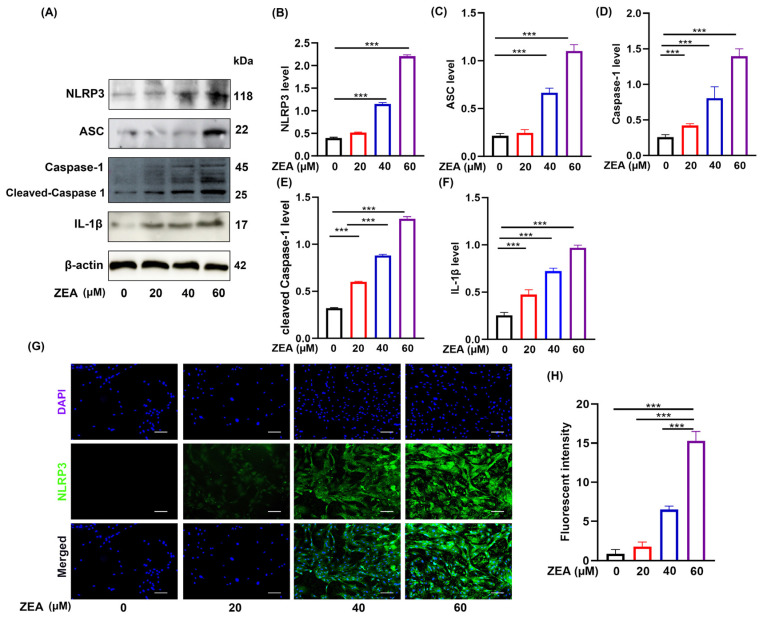
ZEA activates the NLRP3 inflammasome, which mediates gap junction protein downregulation in GCs. (**A**) Western blotting analysis of NLRP3, ASC, Cleaved Caspase-1, Caspase-1, IL-1β, and GPR30 protein expression levels after ZEA treatment at different concentrations (*n* = 3). (**B**–**F**) Quantitative analysis of NLRP3, ASC, Cleaved Caspase-1, Caspase-1, IL-1β, and GPR30 protein expression levels. (**G**,**H**) Immunofluorescence staining intensity and quantification of NLRP3; Scale bar = 100 μm. The loading control was β-actin, and data are presented as mean ± SD. *** *p* < 0.001. Original images can be found in Supplementary Materials.

Environmental pollutants frequently initiate cellular inflammatory responses via the induction of oxidative stress. Notably, the NLRP3 inflammasome has been identified as a central signaling hub that orchestrates inflammatory activation in ovarian cells. To elucidate the underlying mechanisms by which ZEA impairs gap junctions, we profiled the expression of NLRP3 inflammasome-related proteins. Our findings revealed that ZEA exposure significantly upregulated the protein levels of NLRP3, ASC, Cleaved Caspase-1, Caspase-1, and the downstream pro-inflammatory cytokine IL-1β (Figure 2A–F). Moreover, immunofluorescence analysis corroborated these results, displaying a markedly enhanced fluorescence intensity of NLRP3 in the ZEA-treated group relative to the control (Figure 2G,H).

### 3.3. MCC950 Targets the NLRP3 Inflammasome to Alleviate ZEA-Induced Gap Junction Injury in Ovine Ovarian GCs

**Figure 3:**
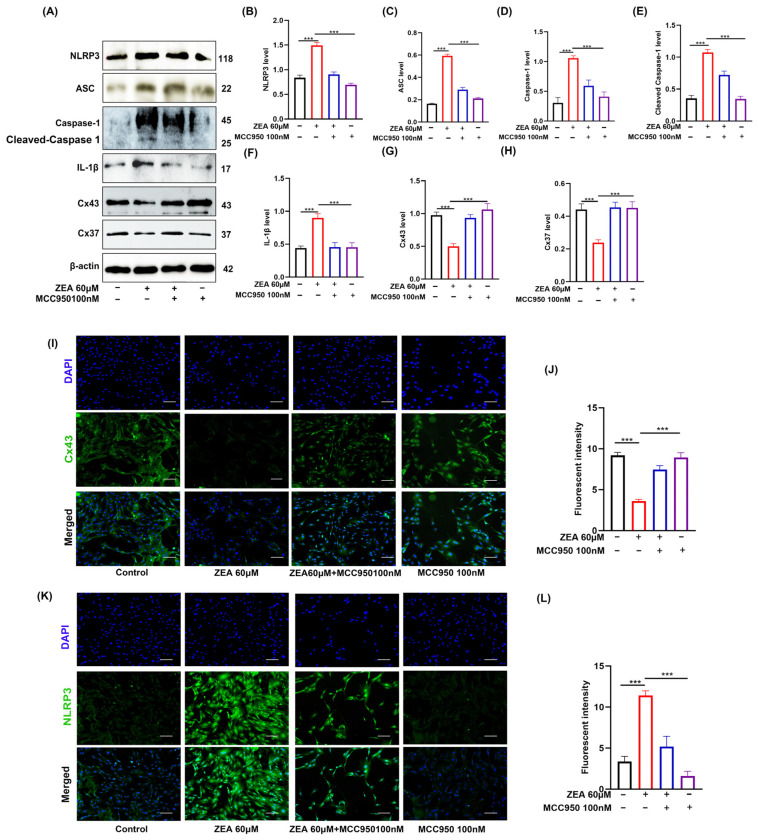
Pharmacological inhibition of NLRP3 with MCC950 restores ZEA-induced downregulation of Cx43 and Cx37 in GCs. (**A**) Western blotting analysis of NLRP3, ASC, Cleaved Caspase-1, Caspase-1, IL-1β, Cx43, Cx37 protein expression levels (*n* = 3). (**B**–**H**) Quantitative analysis of NLRP3, ASC, Cleaved Caspase-1, Caspase-1, IL-1β, Cx43, and Cx37 protein expression levels. (**I**,**J**) Immunofluorescence staining intensity and quantification of Cx43; Scale bar = 100 μm. (**K**,**L**) Immunofluorescence staining intensity and quantification of NLRP3; Scale bar = 100 μm. The loading control was β-actin, and data are presented as mean ± SD. *** *p* < 0.001. Original images can be found in Supplementary Materials.

To further investigate the role of the NLRP3 inflammasome in ZEA-induced gap junction impairment, we utilized MCC950, a specific NLRP3 inhibitor. The results revealed that pretreatment with MCC950 significantly reversed the ZEA-induced downregulation of Cx43 and Cx37 proteins, and markedly suppressed ZEA-induced Caspase-1 activation and IL-1β maturation (Figure 3A–L).

### 3.4. ZEA Disrupts Gap Junctions in GCs by Triggering NLRP3 Inflammasome Activation via ROS Accumulation

**Figure 4:**
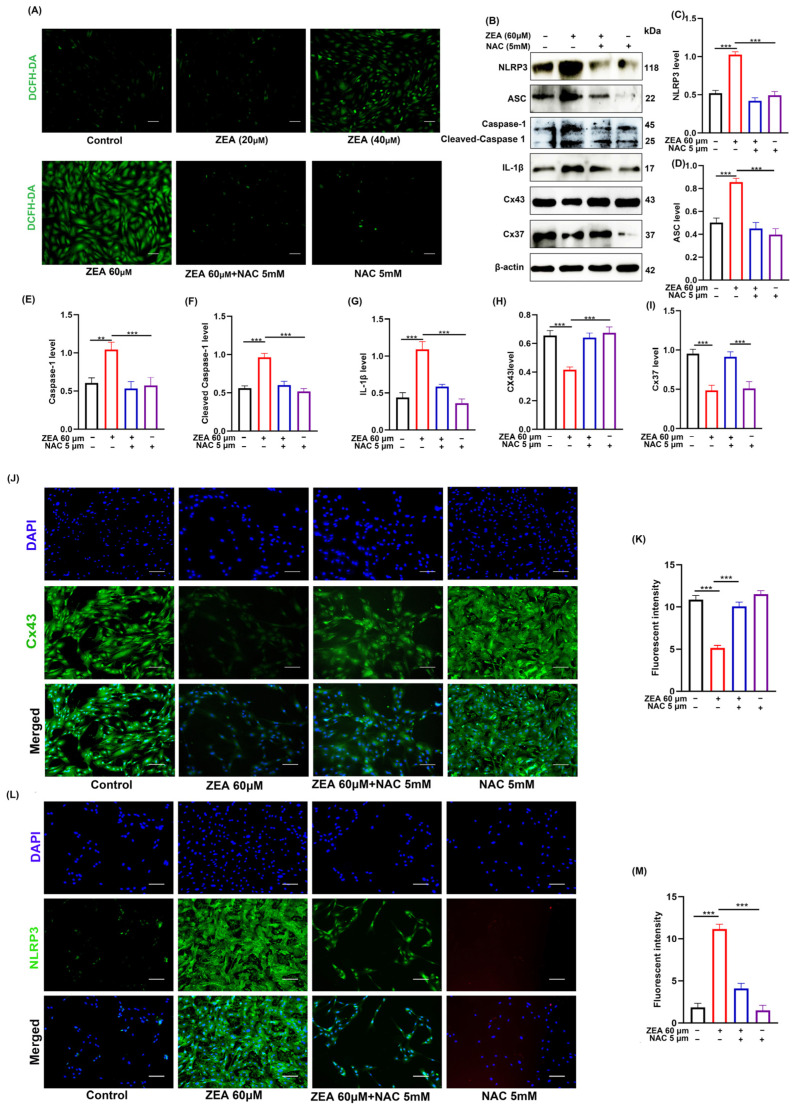
ROS scavenger NAC suppresses ZEA-induced oxidative stress and NLRP3 inflammasome activation, rescuing Cx43 and Cx37 expression in GCs. (**A**) DCFH-DA fluorescence staining to detect ROS levels in GCs after treatment with different concentrations of ZEA. (**B**) Western blotting analysis of NLRP3, ASC, Cleaved Caspase-1, Caspase-1, IL-1β, Cx43, and Cx37 protein expression levels (*n* = 3). (**C**–**I**) Quantitative analysis of NLRP3, ASC, Cleaved Caspase-1, Caspase-1, IL-1β, Cx43, and Cx37 protein expression levels. (**J**,**K**) Immunofluorescence staining intensity and quantification of Cx43; Scale bar = 100 μm. (**L**,**M**) Immunofluorescence staining intensity and quantification of NLRP3; Scale bar = 100μm. The loading control was β-actin, and data are presented as mean ± SD. ** *p* < 0.01, *** *p* < 0.001. Original images can be found in Supplementary Materials.

To investigate the regulatory role of ROS in ZEA-induced NLRP3 inflammasome activation and gap junction impairment in GCs, the oxidative stress inhibitor NAC was utilized. The results demonstrated that ZEA treatment significantly elevated intracellular ROS levels in ovine ovarian GCs, an effect that was effectively reversed by NAC (Figure 4A). Furthermore, NAC pretreatment not only abrogated the ZEA-induced upregulation of NLRP3 and activation of Caspase-1 but also restored the expression of Cx37 and Cx43 (Figure 4B–M).

### 3.5. The GPR30 Receptor Mediates ZEA-Induced Disruption of Gap Junctions in Ovine Ovarian GCs

**Figure 5:**
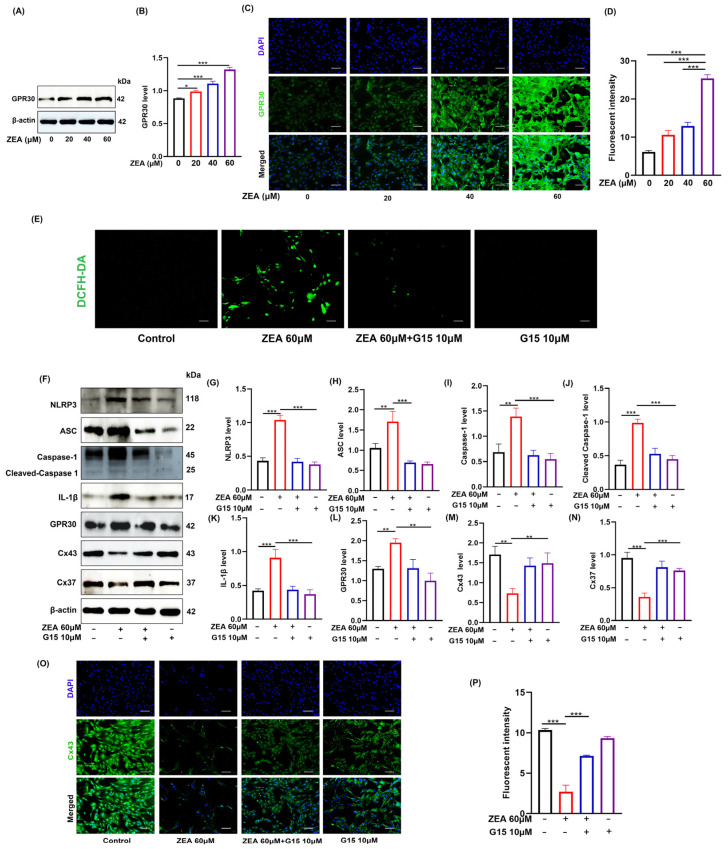
GPR30 antagonist G15 blocks ZEA-induced ROS production, NLRP3 activation, and gap junction protein downregulation, confirming GPR30 as an upstream mediator in GCs. (**A**) Western blotting analysis of GPR30 protein expression levels (*n* = 3). (**B**) Quantitative analysis of GPR30 protein expression levels. (**C**,**D**) Immunofluorescence staining intensity and quantification of GPR30; Scale bar = 100 μm. (**E**) DCFH-DA fluorescence staining to detect ROS levels in GCs. (**F**) Western blotting analysis of NLRP3, ASC, Cleaved Caspase-1, Caspase-1, IL-1β, GPR30, Cx43, and Cx37 protein expression levels (*n* = 3). (**G**–**N**) Quantitative analysis of NLRP3, ASC, Cleaved Caspase-1, Caspase-1, IL-1β, GPR30, Cx43, and Cx37 protein expression levels. (**O**,**P**) Immunofluorescence staining intensity and quantification of Cx43; Scale bar = 100 μm. The loading control was β-actin, and data are presented as mean ± SD. * *p* < 0.05, ** *p* < 0.01, *** *p* < 0.001. Original images can be found in Supplementary Materials.

Finally, we examined whether GPR30 is involved in the above-mentioned effects of ZEA.The results revealed that ZEA treatment altered GPR30 protein expression levels(Figure 5A–D). Pretreatment with G15, a specific GPR30 antagonist, significantly attenuated the ZEA-induced accumulation of ROS (Figure 5E). Concurrently, G15 pretreatment abrogated the ZEA-induced upregulation of NLRP3 and activation of Caspase-1, as well as the downregulation of Cx43 and Cx37 (Figure 5F–P). Collectively, these findings suggest that GPR30 acts as an upstream regulator in this cascade, mediating the ZEA-induced activation of the ROS/NLRP3 signaling axis and the subsequent impairment of gap junctions.

## 4. Discussion

Increasing evidence indicates that mycotoxin contamination of agricultural products poses potential threats to ecosystems and animal health, making it a major global public health concern [26]. Among these mycotoxins, ZEA has garnered widespread attention due to its estrogen-like activity and toxic effects on the animal reproductive system. Previous studies have shown that ZEA induces apoptosis in porcine GCs through the mitochondrial pathway [18]. In chicken follicular cells, ZEA triggers apoptosis by activating the PI3K–AKT–mTOR and MAPK signaling pathways, and simultaneously induces autophagy as a cytoprotective response [27]. In mouse GCs, ZEA primarily exerts its effects via the mitochondrial apoptotic pathway [28]. However, these studies have predominantly focused on apoptosis, and whether ZEA impairs intercellular communication in GCs through inflammatory pathways remains unclear. The gap junction proteins Cx43 and Cx37 are core components of the intercellular communication network in the ovary. Cx43 primarily mediates communication between granulosa cells, whereas Cx37 is responsible for the coupling between GCs and oocytes; together, they regulate follicular growth and ovulation [29]. In the present study, we found that ZEA exposure inhibits the viability of ovine ovarian GCs and downregulates the expression of Cx43 and Cx37 in a concentration-dependent manner via the GPR30–ROS–NLRP3 signaling axis. Collectively, our findings reveal for the first time a novel mechanism underlying ZEA-induced reproductive toxicity, which involves inflammasome activation and impairment of intercellular communication.

The NLRP3 inflammasome plays a critical role in ZEA-induced gap junction impairment. It has been reported that ZEA induces apoptosis in bovine ovarian GCs through the NLRP3 pathway [30]. In the present study, we further found that ZEA treatment significantly activated the NLRP3 inflammasome, as evidenced by the upregulation of NLRP3, ASC, and Cleaved Caspase-1 expression, as well as the maturation and release of IL-1β, indicating that the NLRP3 inflammatory pathway was activated. To verify whether NLRP3 serves as a key upstream event in ZEA-induced gap junction impairment, we used MCC950, a specific inhibitor of NLRP3. The results showed that MCC950 not only inhibited Caspase-1 cleavage and IL-1β maturation but also significantly restored the expression levels of Cx43 and Cx37. Collectively, our results demonstrate that NLRP3 inflammasome activation is a critical intermediate event in ZEA-induced gap junction impairment.

Oxidative stress acts upstream of NLRP3 inflammasome activation [29]. Excessive ROS serve as a danger signal that triggers the assembly and activation of the NLRP3 inflammasome [31]. In the present study, ZEA treatment significantly induced ROS overproduction in ovine GCs. To further clarify the upstream–downstream relationship between ROS and NLRP3, we performed intervention experiments using the ROS scavenger NAC. The results showed that NAC pretreatment not only effectively reduced ROS levels but also abrogated ZEA-induced NLRP3 activation and significantly restored the expression of Cx43 and Cx37. Collectively, these findings indicate that ROS acts upstream of the NLRP3 inflammasome, and that scavenging ROS can block the initiation of inflammatory signals, thereby protecting gap junction function.

GPR30, acting as the membrane receptor for ZEA, functions upstream of ROS signaling. As a non-classical estrogen receptor localized on the cell membrane, GPR30 serves as a key mediator of rapid signal transduction induced by environmental estrogens [23]. Studies have shown that GPR30 not only participates in follicle-stimulating hormone signaling but is also closely associated with the proliferation, differentiation, and steroid hormone synthesis of granulosa cells. In the present study, ZEA treatment was found to alter the expression levels of GPR30 in GCs. Notably, the specific antagonist G15 of GPR30 significantly blocked ZEA-induced ROS accumulation, NLRP3 inflammasome activation, and downregulation of Cx43/Cx37 expression. These findings provide strong evidence that ZEA acts as an agonist of GPR30, initiating downstream toxic signaling through functional activation of this receptor, rather than solely through changes in GPR30 protein abundance.

Although the present study demonstrates that ZEA impairs gap junction protein expression in ovine GCs via the GPR30/ROS/NLRP3 signaling axis, several limitations should be acknowledged, including the use of ZEA concentrations above environmental levels, reliance on pharmacological inhibitors with potential off-target effects, inference of gap junction dysfunction solely from protein expression without direct functional assays, and the inability of the in vitro setting to fully replicate the in vivo microenvironment. Future studies using lower concentrations with prolonged exposure and in vivo animal experiments are therefore warranted to confirm the physiological relevance and pathological role of this pathway.

In summary, this study elucidates a novel molecular mechanism by which ZEA induces injury in ovine GCs. ZEA activates GPR30 on the cell membrane, leading to excessive ROS accumulation, which, in turn, triggers NLRP3 inflammasome activation and downregulates the expression of gap junction proteins, ultimately impairing intercellular communication. These findings not only expand our understanding of ZEA-induced reproductive toxicity but also identify GPR30 and NLRP3 as potential therapeutic targets, providing an important theoretical basis for the prevention and treatment of mycotoxin-induced ovarian damage.

## 5. Conclusions

This study is the first to reveal a novel mechanism by which ZEA induces inflammation in ovarian granulosa cells and damages ovarian gap junctions via the GPR30–ROS–NLRP3 signaling axis, providing a new perspective for understanding the reproductive toxicity of ZEA.

## Data Availability

The original contributions presented in this study are included in the article; further inquiries can be directed to the corresponding author.

## Supplementary Materials

The following supporting information can be downloaded at: https://www.mdpi.com/article/10.3390/biom16060837/s1.
