# Supplementary material for: Zearalenone Induces Gap Junction Damage in Ovine Ovarian Granulosa Cells by Upregulating GPR30 and Activating the Oxidative Stress–NLRP3 Inflammasome Axis
Source: Biomolecules. 2026 Jun 7;16(6):837. doi: 10.3390/biom16060837 (PMC13297281; doi:10.3390/biom16060837)
Supplement: Supplementary file 1 [file biomolecules-16-00837-s001.zip › biomolecules-4296749-supplementary.pdf]

Fig 1.

β-actin	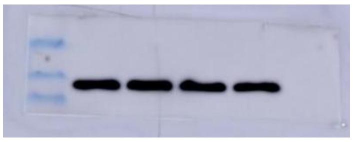
Cx43	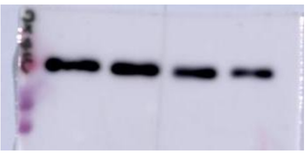
Cx37	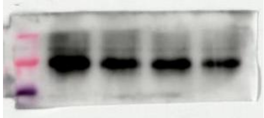

Fig 2.

β-actin	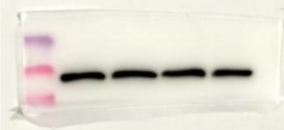
IL-1β	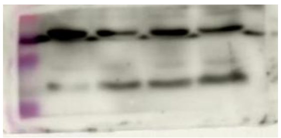
Caspase-1/Cleaved Caspase-1	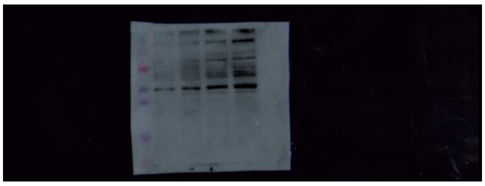
ASC	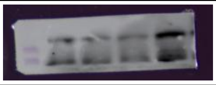
NLRP3	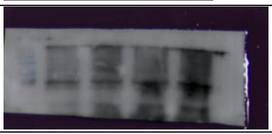

Fig 3.

β-actin	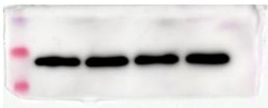
Cx37	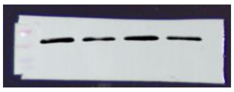
Cx43	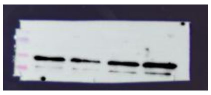

IL-1β	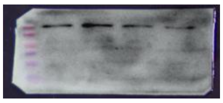
Caspase-1/Cleaved Caspase-1	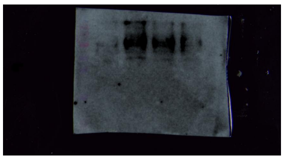
ASC	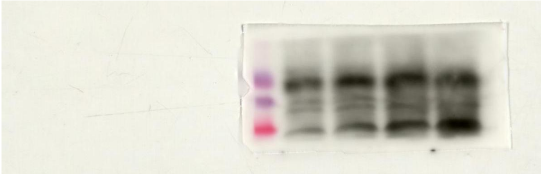
NLRP3	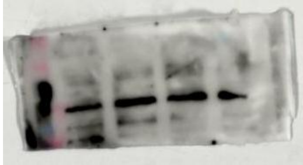

Fig 4.

β-actin	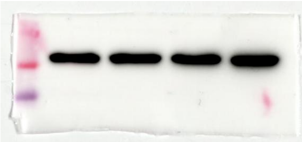
Cx37	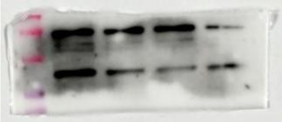
Cx43	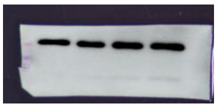
GPR30	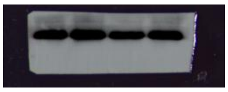
IL-1β	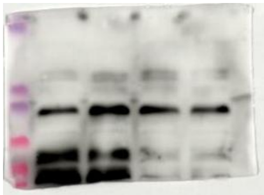
Caspase-1/Cleaved Caspase-1	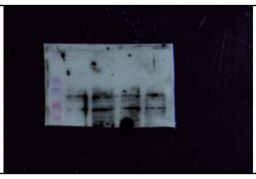

ASC	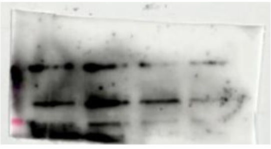
NLRP3	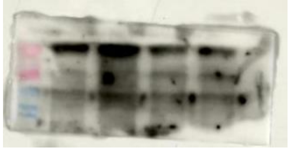

Fig 5.

β-actin	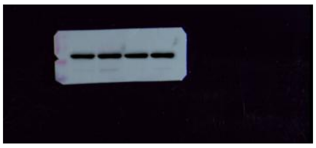
GPR30	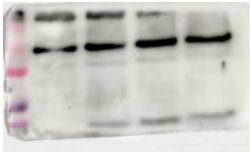
β-actin	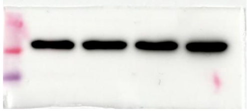
Cx37	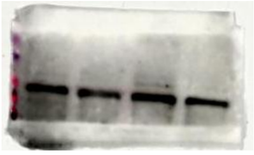
Cx43	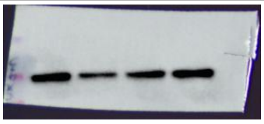
GPR30	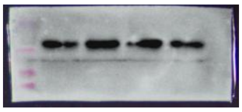
IL-1β	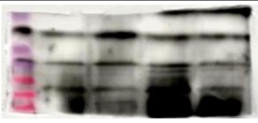
Caspase-1/Cleaved Caspase-1	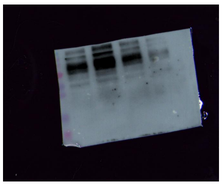
ASC	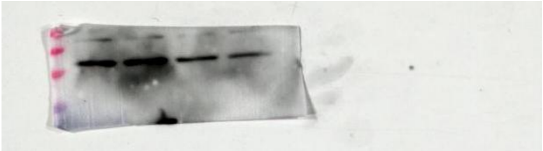

NLRP3	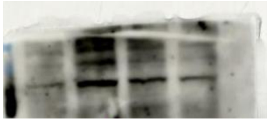
